# Care Practices: A Concept Analysis

**DOI:** 10.3390/nursrep16100363

**Published:** 2026-10-13

**Authors:** Marcio Jiménez-Gutiérrez, Elizabeth Vargas-Rosero, Gloria Mabel Carrillo-González, María de los Ángeles Torres-Lagunas, Guillermina Arenas-Montaño, Irma Cortés-Escárcega, Rosa María Ostiguín-Meléndez

**Affiliations:** 1 Master’s and Doctoral Program in Nursing, National Autonomous University of Mexico (UNAM), Mexico City 04510, Mexico; angelestorres@comunidad.unam.mx (M.d.l.Á.T.-L.); guillearenasm@iztacala.unam (G.A.-M.); icecor2@yahoo.com (I.C.-E.); sec-gral@eneo.unam.mx (R.M.O.-M.); 2 Faculty of Nursing, National University of Colombia (UNAL), Bogota 111321, Colombia; evargasr@unal.edu.co (E.V.-R.); gmcarrillog@unal.edu.co (G.M.C.-G.); 3 Faculty of Nursing and Obstetrics (FENO), National Autonomous University of Mexico (UNAM), Mexico City 14370, Mexico; 4 Faculty of Higher Studies Iztacala (FESI), National Autonomous University of Mexico (UNAM), Tlalnepantla 54090, Mexico; 5 Faculty of Higher Studies Zaragoza (FESZ), National Autonomous University of Mexico (UNAM), Mexico City 09230, Mexico

**Keywords:** care practices, concept analysis, care, nursing theory, practical nursing

## Abstract

**Background/Objectives**: To analyze the concept of “care practices” as an abstract, relevant element of interest to the nursing discipline. **Methods**: The Walker & Avant methodology was followed, a structured eight-stage process that ranges from the choice of the concept and the identification of its essential features to the creation of comparative examples (cases) and the definition of their practical effects. The analysis was based on the paradigm of simultaneity, a vision that breaks with the traditional biomedical model, to understand the human being as an indivisible unit. Recognizing the tension between the analytical-positivist roots of the method and the chosen paradigm, Walker & Avant was used in an adaptive, heuristic way. **Results**: Based on the review of 12 predominantly qualitative articles, “care practices” are defined as actions aimed at solving needs in situations of vulnerability through an interpersonal bond influenced by the environment. Five defining dimensions were identified: awareness of vulnerability, activities in the intersubjective human bond, influence of the environment, recognition of intersectionality without judgment, and the active care of the self. **Conclusions**: Concept analysis provides nursing with scientific rigor, allowing for the delimitation of its scope and grounding its practice. “Care practices” transcend a mere activity to become a profound human encounter based on the recognition of shared vulnerability, involving not only healthcare personnel but human beings in general.

## 1. Introduction

Care represents the fundamental core of nursing as a discipline. This practice can be understood as an endeavor that demands both personal and ethical–professional commitment, oriented toward preserving life, facilitating recovery, and promoting the patient’s autonomy in their own care, all sustained by the therapeutic bond established between the nurse and the person receiving care [1]. However, this practice is not exclusive to, nor the sole interest of, any single professional discipline. Instead, it constitutes a human activity inherently linked to life itself, which responds to the condition of vulnerability—whether one’s own or that of others—and possesses a relational, contextual, non-essentialist, and responsible nature [2]; this aspect explains why care is a phenomenon of interest across the health sciences, social sciences, and moral philosophy, without any of them being able to claim it as their exclusive object of study.

Although care is an inherent professional dimension of nursing, actualized within its practice, it is crucial to analyze this concept through a disciplinary perspective and methodology. This requires transcending its conceptualization as the merely instrumental or mechanical execution of tasks, elevating it to a truly ethical sense founded on interaction, communion, and empathy that places the other at the center [3].

Since Nightingale, care practices have been formalized through methodological rigor and systematic intervention, establishing themselves as a sophisticated epistemological category [4]. This evolution has consolidated care not merely as a humanitarian act, but as a structured disciplinary object of study [5].

However, this disciplinary consolidation does not exhaust the phenomenon, as it is relevant to highlight that care practices are not only an activity exclusive to nursing, but also of interest to other professions and essential for the preservation of humanity: they are also carried out outside the clinical setting—in families, tangible and digital communities, and peer groups—and are traversed by configurations of gender, class, race, age group, and cultural constructions of the body [6] that exceed traditional institutional and healthcare professional logic.

Thus, it is pertinent and necessary to clarify the concept of ‘care practices’, even though they are not exclusively executed by nursing. This allows not only for the strengthening of its disciplinary use but also provides an analytical framework applicable to contemporary phenomena where care emerges in non-conventional contexts—mediated by gender, spaces, corporeality, and culturally situated practices—and where the boundary between caring and harming, between well-being and risk, demands a rigorous conceptualization that no single discipline can sustain in isolation.

### 1.1. Care as a Situated Human Practice, Linked to Vulnerability

Nursing recognizes the centrality of the concept of caring within its disciplinary knowledge structure [5], but the practice of care is neither exclusive nor original to it: it converges with disciplines whose object is human activity, vulnerability, and interdependence, such as anthropology, psychology, sociology, and moral philosophy. From the perspective of the ethic of care, Tronto has argued that care constitutes a relational and political practice, organized into phases—caring about, taking responsibility, caregiving, carereceiving—that articulate affective, material, and power dimensions [2]. Gilligan, for her part, demonstrated that care operates as a moral rationality distinct from the logic of abstract justice, anchored in responding to the concrete vulnerability of the other [7].

In both senses, it is important to highlight a common element: vulnerability as a condition inherent to human beings and a necessary requirement for care. We are all vulnerable and fragile at some point in life, especially during childhood, old age, or illness, but also, less visibly, in everyday projects of bodily transformation, in vital crises, in experiences of exclusion, and in the dependence on peer networks to sustain decisions that affect one’s own body.

Autonomy is not opposed to interdependence: it is its outcome [2]. Vulnerability and need, rather than being viewed as a problem to be eradicated, concealed, or resolved, exist as central elements of life that require mutual attention: understanding care implies, therefore, abandoning the obsession with control and recognizing fragility as a shared condition [2,7].

### 1.2. Brief Genealogy and Dynamism of Care Within the Disciplinary Domain of Nursing

The link between care and vulnerability is grounded in learning, the bodily dimension, and environmental adaptation. Caring implies an interaction with the other that balances personal autonomy, mutual assistance, and the expression of needs. These concepts allow us to trace the evolution of care from prehistory to the present day, analyzing aspects such as archaeological materiality, the perception of ‘normalcy versus abnormality’ in ethnographic groups, rights-and-obligations systems in tribes, and learning during the Paleolithic [8].

Care practices are the result of an evolutionary process where nature and culture are dynamically articulated. Affective bonds are the main driver of constant care, especially in the face of vulnerability. This behavior has deep roots: fossil and archaeological remains exist that demonstrate how these bonds enabled survival in highly hostile prehistoric environments [8].

From the professional context and throughout the 20th century, with the consolidation of nursing as a discipline, care practices were reconfigured from various theoretical currents (humanistic, phenomenological, sociological), transitioning from an instrumental logic toward a relational, ethics-oriented, and person-centered understanding [9]. However, a persistent tension is produced in this process: while care is consolidated as an epistemological category, practices tend to fragment under the influence of the biomedical model and the technical division of labor [10].

Thus, care practices are not neutral: they are historically situated and respond to configurations of power, knowledge, and the organization of care systems [10]. Therefore, this implies recognizing that care practices are also intersubjective, organizational, and political, not merely technical or individual [9]. From an analytical perspective, it can be stated that care practices have transitioned through at least three major moments: (i) a pre-disciplinary moment: care as a social and cultural practice; (ii) a classical disciplinary moment: techno-scientific systematization of care; and (iii) a complex contemporary moment, characterized by the expansion, diversification, and problematization of practices in global, technological, and environmental contexts [11,12].

Currently, the challenge does not lie solely in describing or expanding care practices, but in rearticulating them with the epistemological core of care as an object of nursing. Otherwise, there is a risk that practices may become a set of actions devoid of disciplinary meaning, subordinated to other professional logics [9,11]. That is, care practices are a historical, situated, and dynamic expression of care, whose evolution reflects both the development of nursing and the transformations of health systems. Understanding them demands analyzing them not only as technical work, but as a social, ethical, and epistemological construction in permanent redefinition [10,12].

Furthermore, care is an essential element at the disciplinary level, understanding its place within the holarchic structure of nursing knowledge, whose metaparadigm encompasses the most abstract vision of the discipline within the concepts of person, environment, health, and nursing (care), which constitute a whole in themselves alongside other elements previously considered of lower hierarchy [5]; this vision aligns with the simultaneity paradigm, which conceives the human being as an inseparable unit of their universe, in constant change, and health as a process of becoming rather than a state [13].

In this case, focusing attention on care in its relationship with the empirical and experience (care practices) implies returning to proposals for knowledge development as an emergent, continuous, and human process where practice is not only a field of application, but also of theoretical innovation. This transforms the nurse’s identity from a consumer to a producer of knowledge, considering this practice as the axis of active generation of disciplinary knowledge rather than merely its application; that is, practice as the core of nursing knowledge development [14].

### 1.3. Body, Intersectionality, and Care

The term ‘intersectionality’, introduced by Crenshaw, demonstrated how the categories of gender and race in the legal field are determining factors for oppression and discrimination [15]. However, its use has expanded to encompass the various categories that complicate life in terms of discrimination and privilege, access to rights or barriers that impede their exercise, and the deepening of our understanding of people’s positioning in diverse social contexts and structures [16].

Intersectionality is transversal to care, encompassing gender (domestic and care work linked to the feminine), age components (adults caring for others or the care needs of elderly individuals), racial and migratory aspects (immigrant women caring for native subjects), and other factors [6], making its practice political and social and its requirement variable, as vulnerabilities overlap.

On the other hand, the body is crucial in care practices, where it has been assumed that women—the female body—are exclusively and solely responsible for the care of others, which requires its democratization and the participation of other agents [6]. Meanwhile, for men—the male body—the social construction of masculinity is an obstacle to self-care, as they self-perceive solely as instruments of production (at the service of work or sports), which invalidates their physical exhaustion, the adoption of risks, and the scarce recognition of their own needs [17].

Consequently, incorporating intersectionality and corporeality into the analysis of ‘care practices’ allows for the identification of contexts and particularities that institutional care may ignore, leading to the recognition that the management of vulnerability does not need to be homogeneous, and to an understanding of how the construction of identity and the body become determining aspects in care, transcending the traditional clinical and biomedical spaces that assert neutrality in caring.

### 1.4. Care of the Self: Tension Between Freedom and Control

Although the need for interdependence—derived from the shared condition of vulnerability and its recognition in the other [2,7]—has been assumed, it is essential to consider the caregiver figures who execute care practices in a way that is neither unidirectional nor limited to an instrumental and provision-oriented character [16]. This implies that those who care must also be recipients of care from others, of care of the self, and of the capacity for self-reflection in their actions.

Foucault’s shift from the analysis of power to ‘practices of the self’ and ‘care of the self’ (*Epimeleia Heautou*) represents an ethical and freedom-oriented proposal where care of the self is positioned as ethically primary, since the relationship with oneself is ontologically prior to the relationship with others [18].

This aspect of care involves not only the deployment of ‘technologies of the self’, but a general attitude toward oneself, toward others, and toward the world that entails the exercise of freedom and voluntary actions to make one’s own life a work of art [19].

This crucial and ambiguous aspect, which involves the government of the self and the attitude toward oneself, is relevant in care practices that transcend the aforementioned ‘technologies of the self’ [20], as they do not always align with the guidelines of health institutions in terms of ‘normalization’—which is merely disciplinary and tied to Biopolitics [21,22], understood as the control, management, and optimization of biology and the body—instead becoming a practice of freedom and responsibility that shapes life itself rather than strictly obeying a mandate of the medical model that demands blind obedience.

Furthermore, the biomedical model assumes that the caregiver (professionally or not) is reduced to a technical instrument of inexhaustible energy; therefore, care of the self breaks with the fallacy of the vertical hierarchy upon which this model rests and dismantles the perpetuation of roles between an active subject—fictitiously invulnerable caregivers or health professionals—and a passive one—implying that the care recipient actually possesses autonomy and will—transforming it into a dynamic interaction. This allows for a transition from a superficial strategy aimed at ‘performing more and better’ to a practice of freedom where caring for the other is a decision whose driving force is not sustained by the demands of health institutions, leading to action through inertia.

From this perspective—which departs from the biomedical framework and medical hegemony—care practices are reconfigured from the experiences of the actors themselves toward themselves, others, and the world, serving even as a space of resistance that responds more to a logic of will, relationality, and social connection, rather than solely to the mandates of institutional normativity. Care of the self is an ontological, ethical, and radically necessary condition for the practice of care; it requires the recognition of one’s own fragility and vulnerability in order to understand and accompany the other, acting as a biopolitical resistance that refuses the gears of the disciplinary machinery of health institutions by reclaiming subjectivity.

## 2. Materials and Methods

### 2.1. Concept Analysis Method

A concept analysis was conducted according to the Walker & Avant method, which consists of [23]: (i) selecting a concept; (ii) determining the aims or purposes of the analysis; (iii) identifying all uses of the concept; (iv) determining the defining attributes; (v) identifying a model case; (vi) identifying borderline, related, and contrary cases; (vii) identifying antecedents and consequences; and (viii) defining empirical referents.

This analysis was developed within the framework of the simultaneity paradigm [13], which conceives the human being as a unitary being inseparable from the universe, in constant change, focusing on lived experience, health as a process of becoming, and human–environmental care, thus transcending traditional biomedical approaches [24].

As a methodological clarification, and to preempt paradigmatic critiques regarding the friction between the positivist roots of the Walker & Avant method and the interpretivist nature of the simultaneity paradigm, the analytical-deconstructive roots that are the object of criticism from within the discipline itself are acknowledged, specifically regarding the assumption of fixed conceptual essences as well as a linear relationship between antecedents and consequences [25,26,27]. Although these assertions generate tension within the framework of the simultaneity paradigm, Walker & Avant’s methodology was used strictly as a heuristic adaptation and a procedural map to organize the systematization of the analysis, rather than as a rigid positivist tool. Meanwhile, the interpretation of the attributes, antecedents, consequences, and empirical referents was conducted from a non-causal and non-deconstructive reading. In this sense, antecedents and consequences are not understood as a temporal-causal sequence, but rather as conditions that coexist and mutually redefine each other in the concrete practice of care. Therefore, the empirical referents are mostly understood and interpreted as descriptions and narratives of lived experiences rather than as attributes or variables susceptible to measurement and statistical processing.

### 2.2. Data Sources

A systematic literature review on care practices was conducted in accordance with the PRISMA 2020 guidelines [28] up to April 10, 2026, consulting the following databases with no date range restrictions: PubMed, Web of Science, Scopus, SciELO, and LILACS, using the following search strategy: ((((((“care practice”[Title/Abstract]) AND (“vulnerability”[Title/Abstract])) NOT (“professional”[Title/Abstract])) NOT (“nursing”[Title/Abstract])) NOT (“medical”[Title/Abstract])) NOT (“review”[Title/Abstract])), adapted to the syntax of each database. The exclusion of the terms nursing, professional, and medical was a deliberate decision, consistent with the study’s paradigmatic and conceptual perspective, given that care practices also emerge in community, family, peer, and digital contexts—outside the professional healthcare framework. This strategy seeks to capture the concept in its broad sense and avoid saturation by institutional and clinical literature that biases conceptualization toward the biomedical model. This decision introduces, as a trade-off, the exclusion of specific disciplinary nursing production, a limitation that is addressed by engaging in dialogue with said literature in the theoretical-conceptual foundation and discussion sections.

The inclusion criteria were therefore original articles that theoretically or empirically addressed care practices in situations of vulnerability; qualitative, quantitative, or mixed-methods studies; published in peer-reviewed journals. Systematic reviews, editorials, letters to the editor, and articles without full-text access were excluded. The studies finally included in the analysis span the period 2002–2025.

### 2.3. Data Analysis

Two researchers independently screened the titles and abstracts of the references. Any discrepancies during the study selection and analysis phases were resolved through consensus meetings involving the two primary researchers. When an agreement could not be reached, a third researcher intervened to make a final decision based on a joint re-evaluation of the established inclusion and exclusion criteria. The full texts of the studies were reviewed following the same methodology. Two researchers extracted and analyzed each article included in this study to determine the definition of the concept ‘care practices’ and to identify its attributes, antecedents, and empirical referents.

## 3. Results

**Figure 1:**
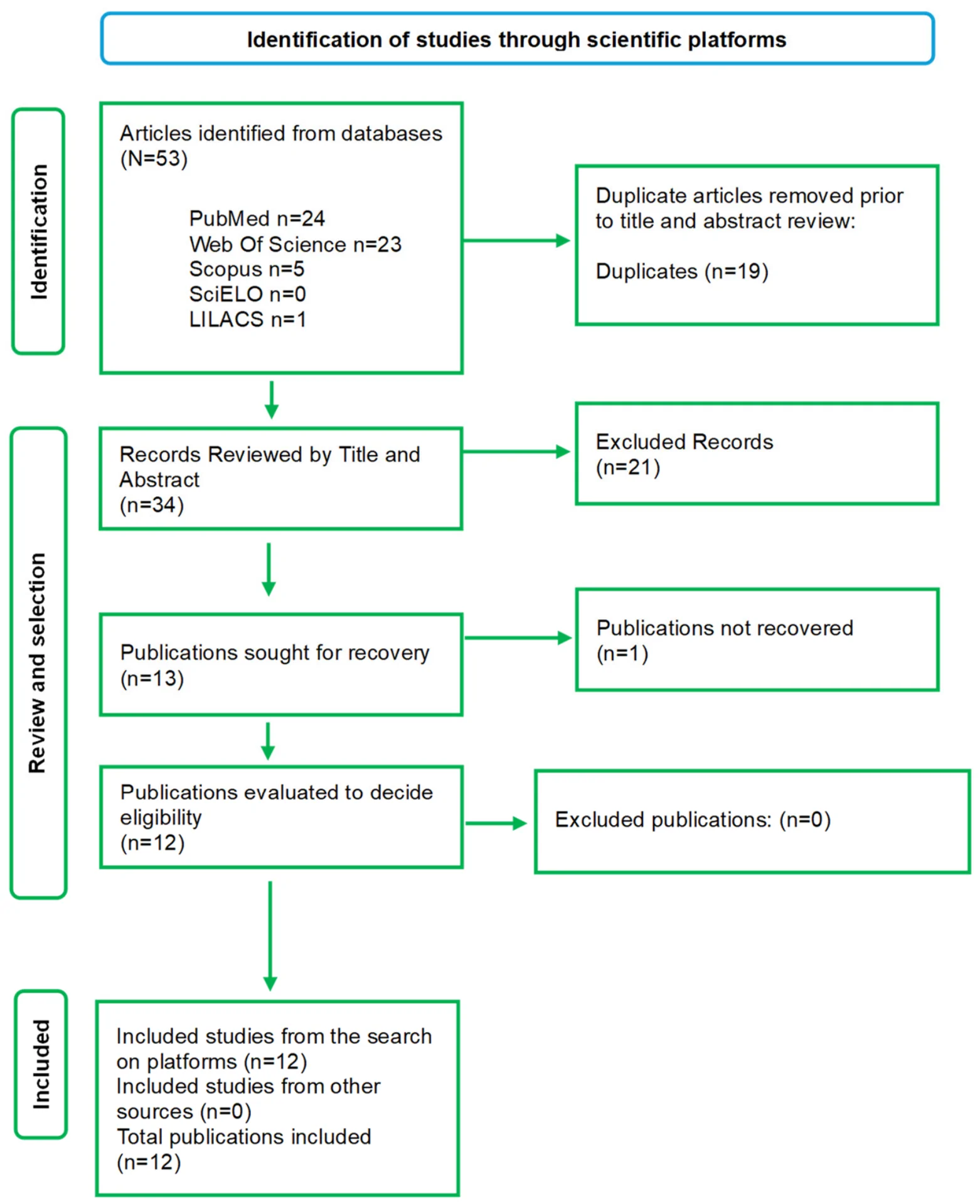
PRISMA 2020 Flow Diagram of the Article Selection Process for the Concept Analysis.

The initial search yielded 53 articles, from which 19 duplicates were removed, leaving 34; after screening titles, abstracts, and full texts, 12 studies were included in the conceptual analysis. Aligned with an analysis that goes hand in hand with the simultaneity vision of the profession, ten of them were qualitative, while one was mixed-methods and another quantitative (descriptive cross-sectional). Uses of the concept, its defining attributes, the model case, borderline, related, and contrary cases, its antecedents, consequences, and empirical referents were analyzed—the latter being mostly narratives that gave voice to the study participants. Figure 1 illustrates the study selection process according to the PRISMA flow diagram. The analytical details per study are available in the Supplementary Materials.

### 3.1. Uses of the Concept of ‘Care Practices’

In a broad sense, it can be inferred that care is the attention to and resolution of the needs of another person or group, within the context of a relationship, that encompasses vulnerability [2,7].

The uses of the concept are extensive; however, stemming from the methodology employed, **care practices** can be considered as those actions—whether professional or non-professional—intended to resolve needs associated with vulnerability (illness, lack of expertise, extreme ages, end of life, and functional diversity) within the framework of interpersonal relationships and under the influence of the environment in which they take place [29,30,31,32,33,34,35,36,37,38,39,40].

### 3.2. Defining Attributes

Based on the methodology employed, the following attributes derived from the analysis of the selected literature were identified:

1. Awareness of the vulnerability, fragility, and imperfection of the other: this implies accepting the human condition of fragility and change, recognizing the interdependence of human beings, and fostering responsibility and solidarity [29,32,33,36,37,39,40].
2. Activities that meet the needs of another within the framework of human relationships: care practices are not limited to the execution of tasks, but entail presence, understanding, and empathy; these activities can involve an intersubjective process of recognizing the emotions and perspective of the other [31,33,34,37,39].
3. Influence of the environment in which they are executed: organizations, institutions, and culture have a bearing on care practices [30,34,38,39,40].
4. Recognition of intersectional categories without judgment or stigma: care practices do not devalue or discredit individuals based on specific aspects, recognizing the categories that explain the human condition, such as race, social class, gender, sexual identity, and functional diversity, among others; that is, they understand and embrace intersectionality [33,38,39,40].
5. Active care of the self (of the care provider): Rather than a prior state, it is the ongoing ethical positioning and recognition of one’s own fragility that allows the provider to be fully present [30].

### 3.3. Identification of a Model Case

#### Model Case

Sofía, an 18-year-old woman experiencing homelessness, is accompanied by her best friend Ximena, who is in the same situation, to a health institution with the objective of legally terminating her 10-week pregnancy. Sofía expresses that she always desires Ximena’s company and support during such a crucial decision and moment; therefore, the health personnel, validating this request despite it being contrary to regulations, allow the access and proximity of the companion. In addition to providing proper care, follow-up, and attention free from judgment and stigmatization, as well as the medication regimen with mifepristone and misoprostol, she is referred to other specialists to connect her with family support networks and non-governmental organizations in order to restructure her social condition and dignity, both for her and her companion.

Attributes present: This case encompasses all five defining dimensions. The triple vulnerability of Sofía—youth, homelessness, reproductive decision—and the shared vulnerability of Ximena are recognized (1); the actions are embedded in an intersubjective bond that acknowledges the presence of the companion as a constitutive part of care (2); the institutional environment enables and sustains the practice by adapting its protocols (3); care is offered without moral judgment regarding the decision to terminate the pregnancy or the situation of homelessness, explicitly articulating intersectional categories (4); the flexibility of the institutional response is also the result of the care of the self-embodied in the health personnel, deriving from a government of themselves in the face of their own values, without blind obedience to regulations, integrating support networks through the recognition of interdependence, accepting the limits of their technical and emotional actions, and decentralizing care (5).

### 3.4. Identification of Borderline, Related, and Contrary Cases

#### 3.4.1. Borderline Case

Jorge, a 21-year-old cisgender man who is part of the LGBTIQ+ community, wishes to take a rapid HIV antibody test. Initially, he asks a relative where he can request such a service; however, the response he receives is one of rejection and stigmatization, as he is prejudged for having unprotected sex, only to later be referred to a specialized center. Upon attending the sexually transmitted disease testing center, during the consultation, the health personnel perform the rapid test, which yields a negative result. However, he is questioned about his sexual orientation and receives no further information regarding his options for HIV prevention, such as condom use and PrEP (Pre-Exposure Prophylaxis).

Attributes partially present: Jorge receives the technical component of care and accesses the institutional environment (attribute 3, present), but the relational dimension (2) and the judgment-free intersectional dimension (4) are compromised: the questioning regarding his sexual orientation and the omission of specific preventive information for his profile demonstrate that the recognition of his vulnerability, both internal and external (1, 5), is incomplete. Given the simultaneous coexistence of present and absent attributes, the case operates as borderline rather than contrary.

#### 3.4.2. Related Case (Technical Efficiency)

Luis, a nurse, works in a surgical inpatient unit. He is in charge of ten patients and is known for his impeccable punctuality. He enters room 302 to administer an intravenous antibiotic to Mrs. Carmen. Luis enters looking at the nursing sheet, checks the patient’s identification bracelet, informs Mrs. Carmen of the procedure, verifies the prescription as well as her allergy history, and without saying another word, proceeds to disinfect the IV port. Luis finishes hanging the bag, notes the exact time in the record, and leaves the room for the next bed, leaving Mrs. Carmen in silence, staring at the ceiling.

Attributes present/absent: This case shares the institutional dimension (3) and a technical recognition of the clinical condition pointing to vulnerability (1) with care practices, but it lacks the intersubjective dimension (2), which is the relational core of care. Therefore, it operates as a related case—close to the concept in its procedural surface—and not as a full instance of it. It accurately illustrates the instrumental drift that disciplinary literature has identified as a permanent risk in nursing practice under the biomedical model.

#### 3.4.3. Contrary Case

A heterosexual cisgender man, voluntarily and due to his desire to enter a bodybuilding competition, has exogenously self-administered anabolic steroids (testosterone and boldenone) for 10 weeks, following a regimen he saw on social media by an influencer. During week 11 of self-administration, due to the skin manifestations (eruptions) he has developed, his peers at the gym begin to mock him and label him as “doped,” which generates shame and a feeling of “being singled out.” Feeling worried about the repercussions, he goes to a primary healthcare center where a family physician scolds him during the consultation, stigmatizes him, and emphasizes that there is a possibility of hypogonadism and other negative effects due to his actions.

Attributes absent: This case exemplifies the simultaneous negation of all five dimensions of the concept. The vulnerability underlying his pursuit of bodily transformation is not recognized (1); there is no empathic or intersubjective relationship between peers or with the health professional (2); the environment—both the peer setting and the institutional healthcare setting—operates as an instance of stigmatization rather than support (3); the medical response is shaped by moral judgment regarding his decisions, without considering the intersectional configurations (masculinity, bodily ideals, digital mediation) that constitute his practice (4); the absence of ‘care of the self’ in the care provider leads to a lack of reflection on his hierarchical position of power and results in punitive and disciplinary action, just as the poor recognition of vulnerability by his peers leads to pointing fingers and labeling (5). Due to the full and simultaneous absence of all five dimensions, the case operates as contrary.

### 3.5. Identification of Antecedents and Consequences

According to the methodology employed, antecedents are the prior conditions or events indispensable for the concept to emerge, while consequences are the outcomes or impacts that arise from its presence [23]. Nevertheless, under the unitary transformative reading and within the simultaneity paradigm adopted in this study, antecedents and consequences are not understood as a linear causal sequence, but rather as conditions that coexist and mutually redefine each other within the concrete practice of care. Table 1 presents this systematization, and Table 2 provides a synthesis of the attributes and key elements.

### 3.6. Description of Empirical Referents

In alignment with the simultaneity paradigm, empirical referents emerge predominantly from qualitative studies; therefore, they do not need to be entirely measurable but are rather obtained directly from the lived experiences of the participants through observations, interviews, focus groups, narratives, and documents. Each defining attribute of the concept has identifiable empirical referents:

1. Awareness of the vulnerability, fragility, and imperfection of the other: testimonies in which the person cared for reports feeling “recognized,” “seen,” or “accompanied” in their fragility [37]; narratives from family caregivers of structurally vulnerable populations at the end of life [31]; reflections on one’s own bodily imperfection on social media, where there is evidence of support from communities in digital networks [29].
2. Activities that meet the needs of another within the framework of human relationships and interdependence: descriptions of sustained bodily presence, active listening, and compassion documented in interprofessional training [30]; letters and notes from older adults documenting personalized interaction with their caregivers [31].
3. Influence of the environment in which they are executed: institutional protocols sensitive to specific populations, such as those developed by street clinics in Brazil [38]; narratives from professionals on how organizational overload limits compassion [34].
4. Recognition of intersectional categories without judgment or stigma: testimonies from women with disabilities regarding their double vulnerability [40]; narratives from male survivors of abuse regarding their need for gender-adapted communication [39]; experiences of culturally situated aging in genocide survivors [35]; voices of stigmatized communities receiving harm reduction care [38].
5. Active care of the self in the care provider: The recognition of one’s own fragility and vulnerability, and the continuous attention towards them, which facilitates full presence with the other during the intersubjective encounter [30].

These referents do not exhaust the phenomenon, as due to their situated and simultaneous nature, care practices will continue to reveal new referents in each investigated context and moment, which grants them epistemological openness.

## 4. Discussion

The findings of this study allow for a dialogue with other concepts that have been the subject of disciplinary analysis and that follow the theoretical traditions supporting the conceptual and methodological foundation.

Care Practices and Person-Centered Care: The concept of “Person-Centered Care” describes four fundamental pillars that characterize it: holism, which considers the person as a biopsychosocial and spiritual whole; individualization, which adapts care to the unique needs and preferences of each person; respect, which recognizes the dignity, values, and autonomy of individuals; and empowerment, which fosters participation and shared decision-making [41]. Although there is a consideration of dignity and a recognition of fragility, the concept is framed within institutionalized healthcare practice, unlike “care practices,” which may or may not be grounded in this context. This difference is not trivial: person-centered care is institutionally determined by formalized healthcare practice, whereas care practices also operate within family, community, peer, and digital networks, which, by definition, person-centered care does not encompass. This condition is relevant for rethinking care in scenarios where there is no healthcare institution involved, something increasingly frequent in contexts where individuals assume care and self-care responsibilities mediated by information consumption [42].

Care Practices and Poor Care: Conversely, the concept of “Deficient Care” is described and characterized by imprecise clinical assessments, erratic or maladjusted management of the person’s needs, and unjustified delays in service provision; likewise, it includes critical failures in the execution of treatments or in patient transfer and referral processes [43]. Although it shares similarities with the contrary case of this analysis, it operates through a different logic: deficient care is defined by the presence of technical failures, whereas the absence of care practices is defined by the simultaneous nonexistence of the five defining dimensions. This suggests that care practices are not defined by the absence of harm, omission, or negligence, but rather by the active presence of a relational, bodily, intersectional, free, conscious, present, and responsible configuration, which differentiates them from both procedural correctness and functional biomedical care.

Care Practices and End-of-Life Care: The concept of “End-of-Life Care” is described from a palliative approach that prioritizes the relief of physical and emotional suffering, placing the patient’s well-being and dignity above any curative intention. Being a humanized and compassionate process that does not seek to alter the natural course of passing but rather focuses exclusively on providing comfort and comprehensive accompaniment in the terminal stage [44], it shares similarities with the concept of “care practices” by emphasizing vulnerability at the end of life, intersubjective relationships, and affect. However, the distinction lies in the fact that the former is circumscribed to a specific stage of the life cycle, whereas care practices operate at any time and in any configuration of vulnerability, even in those moments of transient vulnerability that come to be invisibilized or minimized, which broadens the disciplinary scope of the concept without diluting it.

Dialogue with the Theoretical Framework: The five identified dimensions resonate with theoretical traditions that enrich their understanding. The dimension of vulnerability and interdependence dialogues with the phases of care proposed by Tronto [2] and with Gilligan’s ethics of care [7], which have shown how responding to concrete vulnerability constitutes its own moral rationality. The intersubjective dimension is illuminated by Mol’s logic of care [42], which distinguishes relational care from the consumerist logic of individual choice. The judgment-free intersectional dimension is anchored in the feminist embodied ethics of Toffoletti and colleagues [29], who understand care practices in digital environments. The boundary between care and harm that becomes visible in the contrary case finds its theoretical matrix in Foucault’s “technologies of the self” and Crawford’s concept of healthism [45], which show how self-administered practices can be articulated simultaneously as a pursuit of well-being and as sites of biopolitical control.

Contemporary Applications of the Concept: This conceptualization is particularly productive for phenomena where care practices emerge outside conventional institutional frameworks: digital networks mediating bodily ideals and body modification practices, peer communities sustaining health information outside formal healthcare systems, self-administered practices that oscillate between care of the self and self-inflicted harm, and the deployment of “technologies of the self” that currently operate as self-managers in the construction of identities and even as forms of biopolitical resistance for subjectivities and bodies that do not operate under the mandates of the traditional medical model. The utility of the concept resides, precisely, in its capacity to illuminate these territories without imposing a strict healthcare professional framework that might oversimplify their complexity.

Implications for Practice, Education, and Research: In clinical, community, and out-of-hospital practice, assuming the five defining dimensions implies transcending efficiency as the sole criterion of quality and recognizing that presence, bonding, and intersectional sensitivity are just as constitutive of care as technique. In education, training for care requires addressing the student’s care of the self as a formative condition rather than an extracurricular add-on, integrating perspectives on gender, class, race, ableism, and corporeality into the curriculum. To bridge the gap between theoretical care and clinical nursing practice, these five attributes must be directly operationalized. For instance, curriculum design should integrate specific clinical assessment tools and Objective Structured Clinical Examinations (OSCEs) that evaluate not only technical proficiency but also a student’s capacity for intersubjective bonding, intersectional sensitivity, and self-reflective practices during simulated scenarios. This approach allows the development of the capacity to inhabit intra- and out-of-hospital clinical contexts without strictly limiting them to the traditional biomedical paradigm. In research, the concept opens lines of inquiry into care practices within “informal networks” and tangible and digital peer communities, which transform the transmission and practice of contemporary care.

Limitations: This study presents limitations that must be acknowledged. By anchoring the search strategy around the concept of “vulnerability”, the objective was to capture care as a universal human response across various contexts, rather than strictly within clinical environments. However, by deliberately excluding the terms “nursing”, “professional”, and “medical”, the search strategy inevitably left out literature specific to the nursing discipline. Although this exclusion aligns with the study’s paradigmatic and conceptual perspective and was partially offset by theoretical development, it does limit the empirical coverage. Specifically, this approach may have inadvertently omitted nursing literature that documents valuable community-based, alternative, or informal care practices. The geographical, populational, and thematic heterogeneity of the twelve included studies enriches conceptualization; however, it introduces synthesis complexities that were managed through interpretative consensus, which does not eliminate the possibility of alternative readings. The paradigmatic tension between the Walker & Avant method and the lens of the simultaneity paradigm was resolved through an adapted and heuristic use of the method. While this represents a constraint, it equally demonstrates the flexibility of nursing’s disciplinary methodology and its capacity for dialogue with perspectives such as interpretivism, hermeneutics, and qualitative approaches in general. This adaptation is unorthodox and may face methodological objections. Furthermore, the dimension or attribute of “active care of the self” demonstrates limited empirical representation, having been identified in only one primary study. However, under the interpretivist lens of the simultaneity paradigm, the retention of an attribute is not dictated by its statistical frequency in the literature, but by its ontological necessity to define the concept. Drawing on a Foucauldian perspective, *Epimeleia Heautou* (care of the self) is ontologically prior to the care of others; it is the ethical foundation that prevents the caregiver from becoming an unreflective instrument. Omission of this attribute would have inadvertently perpetuated the hierarchical asymmetry of the traditional medical model, which often assumes care providers to be inexhaustible and strictly functional entities. This theoretical retention challenges biomedical hegemony and highlights a critical gap in the empirical literature, underscoring the urgent need for future qualitative studies to center the caregiver’s subjectivity and transcend the unidirectional approach to care practices. Finally, due to the situated and simultaneous nature of the phenomenon, this analysis does not exhaust the concept: care practices will continue to reveal new dimensions in every investigated context, granting the concept a permanent epistemological openness and ensuring an ongoing dialogue with emerging realities.

## 5. Conclusions

The concept analysis method, adapted to the simultaneity paradigm, allows for the clarification and delimitation of complex disciplinary phenomena, the elimination of ambiguities, the distinction of the concept from related categories in other disciplines, and the provision of systematic support for nursing research and practice. The specific contribution of this analysis has been to delimit care practices into five simultaneous dimensions: recognition of vulnerability and interdependence, intersubjective bond, constitutive influence of the environment, recognition of intersectionality without judgment, and the active care of the self. It also distinguishes them from both technical efficiency and deficient care, opening the concept to non-clinical contexts while maintaining its disciplinary foundation.

This analysis opens concrete lines for future research: empirical studies on care practices in digital communities and peer networks; analysis of the boundary between care and harm in contemporary practices; research with a gender perspective regarding the identity construction of masculinity and femininity and its transversality with the constitutive dimensions of the concept; intersectionality in care and the recognition of practices outside the biomedical model and institutionalized healthcare; the exploration of the role of technological mediations in the transmission and transformation of contemporary care practices; and research that transcends the unidirectional approach to care practices and recognizes hierarchical asymmetry and power relations.

Care practices constitute a transversal human phenomenon that exceeds professional healthcare frameworks, without dissolving nursing’s disciplinary interest in them; on the contrary, it reaffirms it as a privileged lens for understanding and systematizing them. They transcend the procedural: they are an encounter born from recognizing our own shared fragility, an empathic presence that adapts to the needs of the other without judgment.

## Figures and Tables

**Table 1:** Antecedents and Consequences of the concept of ‘Care Practices’.

Antecedents	Consequences
Existence of a condition of vulnerability or fragility experienced in a person or group of people who are going through a situation of need (illness, old age, disability or lack of expertise).	Resolution or mitigation of needs, the main consequence being the attention and assistance of what the other could not solve by himself.
Recognition of interdependence; the potential caregiver must accept that human beings are not self-sufficient, and that frailty/vulnerability is inherent to life.	Strengthening of the intersubjective bond; there is mutual recognition, potential empathy and emotional connection that transcends the technical task.
Existence of a human bond or relationship, making visible that there is no practice of care in a vacuum; it requires the encounter of at least two subjectivities that do not require a close relationship, but do require a genuine mutual interest.	Reciprocal growth in the recognition of imperfection and solidarity, both of those who care and the recipient of care, experiencing a process of learning about the human condition.
A previous socio-political, cultural and digital context that frames what is considered a “need” and who is “authorized” or “called” to care, crossed by gender, class, race, socioeconomic level and technological mediations.	Transformation of the environment through the humanization of organizations and culture or, by contrast, evidence of intersectional inequality gaps where vulnerabilities overlap.

**Table 2:** Key Elements of the Antecedents, Consequences, and Elements of the Concept of ‘Care Practices’.

Category	Key Elements
Background	Vulnerability of the other, awareness of interdependence, cultural/social context.
Attributes	Awareness of fragility, activities in relationships, influence of the environment, absence of judgment, care of the self, intersectionality.
Consequential	Needs met, dignity preserved, bond strengthened, impact on the context, culture or organization.

## Data Availability

Dataset available on request from the authors.

## Supplementary Materials

The following supporting information can be downloaded at https://www.mdpi.com/article/10.3390/nursrep16100363/s1, Table S1: Included Studies and literature analysis of the concept ‘Care Practices’.

## Public Involvement Statement

No public involvement in any aspect of this research.

## Guidelines and Standards Statement

This study was conducted and reported in accordance with the PRISMA 2020 (Preferred Reporting Items for Systematic Reviews and Meta-Analyses) guidelines.
