# Supplementary material for: Care Practices: A Concept Analysis
Source: Nurs Rep. 2026 Oct 13;16(10):363. doi: 10.3390/nursrep16100363 (PMC13708283; doi:10.3390/nursrep16100363)
Supplement: Supplementary file 1 [file nursrep-16-00363-s001.zip › Supplementary File.pdf]

Supplementary Materials

Table S1 Included Studies and literature analysis of the concept 'Care Practices'

Authors / Title	Design	Definition	Definition attributes	Background	Consequences	Empirical references
Toffoletti et al. (2025)<br><br>A feminist embodied ethics of social media use: Corporeal vulnerability and relational care practices	Qualitative study with a feminist approach and Judith Butler's notion of embodied ethics	Care practices imply an embodied ethic that transcends individual self-representation and becomes a micropolitical practice	Awareness of the body's imperfection and vulnerability<br><br>Do not judge the body of the other, recognizing their vulnerability<br><br>Body Image Awareness and Mental Health<br><br>Collective support through messages and advice	Idealized overrepresentation of the body; severe judgment of one's own body and the other with an emphasis on individuality	Recognition of imperfection and vulnerability, transparency in the image shown to the public and emphasis on collectivity	Unfiltered image slideshow on social media<br><br>Narratives of recognition of vulnerability and bodily imperfection<br><br>Collective support
Nissim et al. (2019)<br><br>A Qualitative Study of a	Evaluative qualitative pilot study	Caring for others (in chronic and/or terminal illnesses, such as in people with	Stress management<br><br>Full presence	Fatigue, Burn Out, tiredness, lack of empathy and self-care	Recognition of the vulnerability of oneself and others,	Implementation of specific positive actions; development of presence, empathy and a good

Compassion, Presence, and Resilience Training for Oncology Interprofessional Teams		oncological conditions) implies compassion, presence and resilience; it is preceded by taking care of oneself (the caregiver or professional) avoiding fatigue and exhaustion	Adequate relationship with oneself and derived from adequate interaction with others<br><br>Organizational recognition of the value of work		transparent and adequate relationship with others and the environment, support of the environment in the recognition of needs	relationship with those who are cared for and with peers, which translates into positive aspects of caring for oneself and the other
Rytterström et al. (2013)<br><br>Aspects of care culture in municipal care for elderly people: a hermeneutic documentary analysis of reports of abuse	Qualitative, phenomenological hermeneutical study	The assistance (care practices) provided to vulnerable groups (such as older adults, among others) is immersed in the organization's culture of care, where routine is limited to the execution of tasks without emphasis on interpersonal relationships	It satisfies the needs of human beings in the bed of interpersonal relationships, that is, it is personalized	Adequate execution of tasks that meet the requirements of those who need care without the cultivation of interpersonal relationships	Ethics of care that transcends the execution of tasks in the full recognition of the vulnerable other as a person	Letters, writings and notes from older adults to their relatives that document the aspects indicated

Stajduhar et al. (2019)<br><br>An ethnographic exploration of family caregivers experiences providing care for structurally vulnerable populations at the end-of-life	Qualitative ethnographic study with a critical perspective	Care practices are aimed at groups that may be extremely vulnerable such as people at the end of life and in street conditions; this practice can be in non-consanguineous families (Street Family) or by health and/or care professionals	Care practices in contexts of extreme vulnerability are:<br><br>Executed with palliative objectives at the end of life<br><br>In condition of necessity<br><br>It implies decision-making in the lack of autonomy of those who need care	Care is not received by those who need it most<br><br>Hostile environment<br><br>Limit in the practice of care when derived from the lack of legal recognition of the various forms of relationship and family	Developing culturally relevant approaches where care practice is targeted at vulnerable people and adaptable in critical contexts	Data derived from the voice of those who care for people in extreme vulnerability through recorded and analyzed interviews, field notes and participant observation
Buseh et al. (2002)<br><br>Cultural and gender issues related to HIV/AIDS prevention in rural Swaziland: a focus group analysis	Exploratory qualitative study with focus groups	Care practices are aimed at people with stigmatizing conditions (such as HIV/AIDS) where gender is transversal with the latter aspect; culturally rooted stereotypes represent a risk	Care practices are crossed by the different categories that explain the human condition:<br><br>- Gender<br>- Race<br>- Social level<br>- Educational level	Unmet care needs that differ according to the conditions of vulnerable people<br><br>Vulnerability is diverse and tangible in men and women	The practice of care entails a gender policy that strengthens gender equity, empowerment and its adaptation to women and men, which allows changes in the health of	Qualitative data as empirical references of the interviews and focus groups of the participants who narrate their experiences

		to people's health and integrity	- Sexual orientation	Gender stereotypes represent an accentuation and variability of vulnerability	vulnerable people	
Christiansen et al. (2015)<br><br>Delivering compassionate care: the enablers and barriers	Mixed study: qualitative component with individual interviews; quantitative component with questionnaire of 9 closed and open questions	Care practices include compassion, which is understood as the possibility of emotional connection and capacity for empathy, in this case, of health professionals	Care practices are "compassionate" practices influenced by:<br><br>Individual (values) and interpersonal factors (establishing good relationships)<br><br>Organizations influence their proper development (work overload, lack of staff and results-focused approach)	The practice of care carried out in organizations entails barriers that limit compassion and understanding of the other	The practice of care requires an approach at the individual, organizational and leadership levels, prioritizing the emotional well-being of staff and the creation of positive and supportive work environments	Quantitative data: a tool for closed and open questions<br><br>Qualitative data: individual, analyzed, and coded interviews
Teshuva et al. (2014)<br><br>Experiences of ageing and aged	Qualitative study based on social constructivism	In addition to targeting people with vulnerability, care practices	Care practices are aimed not only at covering physical but also emotional needs; they entail	Activities that are undifferentiated without taking into account the life experiences of	Care practices are framed in cultural competencies, which	Data derived from interviews and focus groups of participants and their families.

care in Australia of older survivors of genocide		involve ethical dilemmas regarding respect for autonomy when expressing the desire not to receive formal care (in this case, in survivors with trauma secondary to experiences of genocide)	ethical aspects of respect for autonomy and awareness in those who have lived traumatic experiences	those who are recipients of them or need them	incorporate knowledge about the long-term effects of people's experiences (experiences of extreme trauma)	
Ferreira et al. (2022)<br><br>Quality of life and social vulnerability of older adults in the urban community: A cross-sectional study	Cross-sectional observational descriptive study	Care practices are in greater demand in the existence of social vulnerability and have an impact on quality of life	Care practices are essential to mitigate social vulnerability; vulnerability encompasses age and socioeconomic status aspects	Vulnerability arises when people lack the capacity to cope with or recover from negative impacts derived from structural, social, and contextual conditions	Care practices in contexts of social vulnerability are fundamental actions for the survival and well-being of people or groups that face structural inequalities, such as poverty, exclusion or	Instruments for measuring Quality of Life and Social Vulnerability:<br><br>WHOQOL-BREF (4 domains: physical, psychological, social relationships, environment) and WHOQOL-OLD (6 facets: sensory skills, autonomy, past/present/future activities, social participation,

					lack of access to basic services	death/dying, intimacy)<br><br>Social Vulnerability Index (IVS) — classifies census tracts as low, medium, high, and very high vulnerability
Tranvåg et al. (2015)<br><br>Relational interactions preserving dignity experience: Perceptions of persons living with dementia	Qualitative study with a hermeneutical phenomenological approach	Care practices are a way of preserving the dignity of people with conditions that make them vulnerable	Care practices have an ethical-interactional aspect in several areas: the family, where love is experienced; social support networks, which enable social inclusion and companionship; with health professionals, who provide human warmth and care immersed in a culture of care	Practices that lack relational ethical sense where interpersonal interaction is omitted or not very tangible	Care practices are relational and generate an experience of dignity is preserved when qualities such as love, inclusion, human warmth, and respect are present in relational interactions	Data come from the voice of the participants through semi-structured individual qualitative interviews with modifiable interview guidance
Bombonatti et al. (2022)	Qualitative study with a focus on	Care practices are also carried	The care practices carried out by	Possibility of criminalization	Care practices focus on the	Data derived from interviews with

Street Clinics and the Healthcare of Vulnerable Homeless Communities in Brazil: A Qualitative Study	social research in health	out by health professionals and can be directed to people in extreme vulnerability (with substance use, LGBT population, with racial discrimination and gender violence)	health professionals are free of stigmatization when targeting people in vulnerability, with a focus on harm reduction	and stigmatization of vulnerable groups who, paradoxically, are the ones who require the most care and health care	removal of judgments and social stigmas, in addition to focusing on harm reduction in people with vulnerability; health care institutions and organizations have specific protocols for the care of these special groups	health professionals with experience in health care for vulnerable groups
Teram et al. (2006)<br><br>Towards malecentric communication: sensitizing health professionals to the realities of male childhood sexual abuse survivors	Multidisciplinary Qualitative Study	Care practices involve active listening that validates people's narratives and adapts to the needs of gender	They involve active listening that validates the narrative of those who need care and are vulnerable<br><br>Adapt to needs by gender	There are specific care needs by gender that are not resolved, in this case of men who have lived through particular traumatic experiences	Care practices have a gender focus, which adapts to the needs of those who require them	Data derived from interviews and focus groups of people who lived traumatic experiences in their childhood

Nicolau et al. (2013)<br><br>Mulheres com deficiência e sua dupla vulnerabilidade: contribuições para a construção da integralidade em saúde	Exploratory qualitative study	Care practices are even more necessary when vulnerability is amplified, so you need to recognize specific needs	They are more necessary in doubly vulnerable conditions and must be accessible to such groups.	Care practices are not general but specific, recognizing the conditions that amplify need and vulnerability	Care practices recognize the specific needs of doubly or triply vulnerable groups	Data derived from semi-structured interviews of people with double vulnerability (women with disabilities)
